# Integrated HIV care is associated with improved engagement in treatment in an urban methadone clinic

**DOI:** 10.1186/s13722-017-0084-y

**Published:** 2017-08-22

**Authors:** Claire Simeone, Brad Shapiro, Paula J. Lum

**Affiliations:** 1 Opiate Treatment Outpatient Program, Division of Substance Abuse and Addiction Medicine, Zuckerberg San Francisco General Hospital and Trauma Center, University of California San Francisco School of Nursing, 995 Potrero Ave, Building 90, Ward 93, San Francisco, CA 94110 USA; 2 Opiate Treatment Outpatient Program, Division of Substance Abuse and Addiction Medicine, Departments of Psychiatry and Family and Community Medicine, Zuckerberg San Francisco General Hospital and Trauma Center, University of California San Francisco, San Francisco, CA USA; 3 Division of HIV, Infectious Diseases, and Global Medicine, Department of Medicine, Zuckerberg San Francisco General Hospital and Trauma Center, University of California San Francisco, San Francisco, CA USA

**Keywords:** Methadone treatment, Retention, Viral suppression, HIV, Engagement, Care continuum

## Abstract

**Background:**

Persons living with HIV and unhealthy substance use are often less engaged in HIV care, have higher morbidity and mortality and are at increased risk of transmitting HIV to uninfected partners. We developed a quality-improvement tracking system at an urban methadone clinic to monitor patients along the HIV care continuum and identify patients needing intervention.

**Objective:**

To evaluate patient outcomes along the HIV Care Continuum at an urban methadone clinic and explore the relationship of HIV primary care site and patient demographic characteristics with retention in HIV treatment and viral suppression.

**Methods:**

We reviewed electronic medical record data from 2015 for all methadone clinic patients with known HIV disease, including age, gender, race, HIV care sites, HIV care visit dates and HIV viral load. Patients received either HIV primary care at the methadone clinic, an HIV specialty clinic located in the adjacent building, or a community clinic. Retention was defined as an HIV primary care visit in both halves of the year. Viral suppression was defined as an HIV viral load <40 copies/ml at the last lab draw.

**Results:**

The population (n = 65) was 63% male, 82% age 45 or older and 60% non-Caucasian. Of these 65 patients 77% (n = 50) were retained in care and 80% (n = 52) were virologically suppressed. Viral suppression was significantly higher for women (*p* = .022) and patients 45 years or older (*p* = .034). There was a trend towards greater retention in care and viral suppression among patients receiving HIV care at the methadone clinic (93, 93%) compared to the HIV clinic (74, 79%) or community clinics (62, 62%).

**Conclusions:**

Retention in HIV care and viral suppression are high in an urban methadone clinic providing integrated HIV services. This quality improvement analysis supports integrating HIV primary care with methadone treatment services for this at-risk population.

## Background

Persons living with HIV and unhealthy substance use are often less engaged in HIV care, have higher morbidity and mortality and are at increased risk of transmitting HIV to uninfected partners [1–4]. The HIV Care Continuum describes key steps to achieve HIV treatment success, from diagnosis to linkage to care, retention in care and finally viral suppression [5]. HIV treatment is considered successful when patients are retained in medical care and achieve viral suppression [6]. According to the most recent published data for the United States, an estimated 86% of people living with HIV had been diagnosed, 40% were engaged in care (defined as having had an HIV medical care visit during the four month sampling period), and 30% achieved viral suppression of HIV (defined as HIV RNA < 200 copies/ml) [7]. UNAIDS set the “90-90-90” goal to end the global AIDS epidemic by 2030, whereby 90% of people living with HIV are diagnosed, 90% of those diagnosed access treatment and 90% on treatment have achieved viral suppression [8]. Current global estimates indicate that 53% of people living with HIV are diagnosed, 41% are in care and 32% are virally suppressed [9–11] with persons who inject drugs identified as a key population for screening and treatment intervention.

Samet et al. [12] described the benefits of linking primary medical care with substance use treatment services which included improved patient access to and satisfaction with both types of health services and better patient outcomes through coordination of care. The authors described models for successful centralized care that integrate medical and psychiatric services into substance use treatment settings.

Subsequent studies have described the impact of methadone and buprenorphine treatment of comorbid opioid use disorder on HIV treatment outcomes. Analyses of a cohort of people who inject drugs (PWID) in Vancouver, British Colombia found an association between methadone maintenance treatment and higher rates of antiretroviral initiation, medication adherence, and viral suppression [13, 14]. In France, better antiretroviral adherence was demonstrated among patients who had ceased injecting drugs while prescribed opioid agonist therapy (methadone or buprenorphine) compared with people who continued to inject drugs. In addition, duration of opioid agonist therapy (OAT) was significantly associated with viral suppression [15]. This research suggests the importance of linking HIV primary care with OAT in order to achieve treatment success in this population.

The Opiate Treatment Outpatient Program (OTOP) is a publicly funded methadone treatment program for patients with opioid use disorder at a large safety net hospital in San Francisco. OTOP serves a patient population with high rates of homelessness, poly-substance use and psychiatric co-morbidities. In addition to methadone treatment, OTOP provides onsite opt-out HIV screening, integrated HIV primary care and psychiatric services, directly observed antiretroviral therapy (DAART), and medical and social HIV case management. Throughout 2015, OTOP provided methadone treatment services to 704 individual patients, 11% of whom had HIV infection based on OTOP’s universal opt-out HIV testing procedures. An HIV prevalence of 11% is similar to that among PWID nationally (11%) [4] and in San Francisco (12%) [16].

OTOP patients infected with HIV have the option to receive their HIV primary care at the methadone clinic from an HIV primary care provider, at a large multidisciplinary HIV specialty clinic located in a building adjacent to the OTOP clinic, or at any number of clinics in the San Francisco community. OTOP patients who receive their HIV primary care onsite at OTOP or at the HIV specialty clinic also have the option of receiving their antiretroviral treatments as DAART along with their methadone dose, an adherence support strategy that was associated with improved viral suppression in a 2007 pilot study at OTOP [17].

At our urban methadone clinic, we developed a quality-improvement tracking system to monitor patients along the HIV care continuum in order to evaluate retention in care and viral suppression for our HIV-infected patients. A second goal of our tracking system was to identify patients who did not meet retention and viral suppression criteria and target those patients for interventions in order to improve treatment success. The purpose of this study was to evaluate patient outcomes associated with OTOP’s HIV Care Continuum and explore the relationship of HIV primary care site and patient demographic characteristics with retention in treatment and viral suppression.

## Methods

We reviewed electronic medical record (EMR) data from 2015 for all OTOP patients with known HIV disease (n = 73) including age, gender, race, HIV care sites, HIV care visit dates and HIV viral load. Patients who left OTOP treatment before the final month of the study year (n = 5) or whose medical records were in a different healthcare system and unavailable (n = 3) were excluded, leaving 65 patients in the final analysis.

Retention was defined as having an HIV primary care visit in both halves of the study year. Viral Suppression (VS) was defined as having an HIV viral load <40 copies/ml at the last determination within the study year (2015). Patients who had their most recent viral load prior to 2015 were classified as not meeting viral suppression criteria. Primary care visit dates and viral load values were transformed into dichotomous variables (yes/no) for meeting the indicator criteria. Age was categorized as <45 and ≥45 years old. Race was extracted from the patient profile in the medical record and was collapsed into three categorical variables (African American, Caucasian and other) to allow for statistical analysis with the small sample size. Site of care was also collapsed into three categorical variables (methadone clinic, HIV clinic and community clinics). Using IBM SPSS [18], data were analyzed with Fisher’s exact test. Expedited IRB approval for this study was granted by the University of California San Francisco Committee on Human Research as a retrospective records review without subject contact or consent.

## Results

The study population was mostly male, over the age of 45 and non-Caucasian (Table 1). Among all patients diagnosed with HIV in treatment at OTOP at the end of the study year who were eligible for analysis (n = 65), 50 (77%) met retention criteria and 52 (80%) were virologically suppressed. Viral suppression was significantly higher for women (*p* = .022) and patients 45 years or older (*p* = .034). A larger proportion of patients receiving care at the methadone clinic compared to the HIV specialty clinic or community clinics were retained in care (93 vs. 74 vs. 63%, *p* = .150) and achieved viral suppression (93 vs. 79 vs. 62%, *p* = .164), although these comparisons did not reach statistical significance.

**Table 1:** Outcomes by demographic characteristics

Characteristic	Variable (N)	Retention in care^a^ N (%)		Viral suppression^b^ N (%)	
		Yes	No	Yes	No
Gender	Female (24)	19 (79%)	5 (21%)	23 (96%)*	1 (4%)
	Male (41)	31 (76%)	10 (24%)	29 (71%)	12 (29%)
Age	<45 years (12)	8 (64%)	4 (33%)	6 (50%)*	6 (50%)
	≥45 years (53)	43 (81%)	10 (19%)	43 (81%)	10 (19%)
Race	African American (27)	20 (74%)	7 (26%)	21 (78%)	6 (22%)
	Caucasian (26)	20 (77%)	6 (23%)	17 (65%)	9 (35%)
	Other (12)	11 (92%)	1 (8%)	11 (92%)	1 (8%)
Site of care	Methadone clinic (15)	14 (93%)	1 (7%)	14 (93%)	1 (7%)
	HIV clinic (42)	31 (74%)	11 (26%)	33 (79%)	9 (21%)
	Community clinics (8)	5 (62%)	3 (38%)	5 (62%)	3 (38%)

^**a**^Retention in care = meets criteria of having a primary care visit in 1st and 2nd halves of the study year

^b^Viral suppression = meets criteria of HIV viral load <40 copies/ml at most recent laboratory draw

* Significant relationships for (a) viral suppression and gender (Fisher’s exact test (2-sided), *p* = .022), and (b) viral suppression and age (Fisher’s exact test (2-sided), *p* = .034)

## Discussion

In a quality improvement clinical investigation, we found that HIV-infected patients enrolled in a publicly-funded methadone treatment program in San Francisco had high rates of retention in HIV care and viral suppression, both markers of HIV treatment success along the HIV care continuum. HIV engagement outcomes assessed in 2015 for OTOP patients diagnosed with HIV far exceeded the most recently reported national HIV care continuum data for retention in care (77 vs. 40%) and viral suppression (80 vs. 30%) [7]. This is an encouraging result given the high frequency of homelessness, poly-substance use and psychiatric co-morbidities among OTOP patients and the known negative impact of these psychosocial circumstances on successful HIV treatment [19, 20].

The extent to which integrated HIV and addiction care may play a role in achieving better outcomes along the care cascade and achieving the U.N. targets of 90-90-90 by 2030, is of considerable interest to this study. We measured a 19 and 31% difference, respectively, in retention in care for patients who received their HIV primary care onsite at OTOP (93%) compared to patients who received their HIV care from the large HIV specialty clinic next door (74%) and compared to patients receiving HIV care from other community clinics (62%). Viral suppression in OTOP primary care patients (93%) also was 14 and 31% higher compared to HIV clinic (79%) and community clinic patients (62%), respectively. These notable differences may reflect the “one-stop shopping” convenience of integrated HIV and methadone treatment or patients’ perceptions of OTOP as a less stigmatizing medical home. Most patients visit an opioid treatment program daily for directly observed methadone dosing, which is very likely to improve retention in co-located HIV care. Furthermore, the opportunity for our HIV-infected patients at highest risk for poor medication adherence to receive their treatment through DAART may contribute to OTOP’s high viral suppression rate. In this regard, Rothman et al. [21] found that co-locating HIV treatment in a variety of New York’s substance use treatment programs was acceptable, effective and efficient in delivering HIV care to this high-risk population. Our research similarly suggests that HIV treatment in methadone clinics may have high levels of acceptance and effectiveness for persons living with HIV and opioid use disorders. While the small size of our patient sample required observing very large differences in order to reach statistical significance, our findings suggest that those differences may be much larger than clinically significant differences. A multi-site study with a larger sample size could be conducted to further explore this relationship. Qualitative research that explores factors that influence patients’ choice of where locate their HIV care could also contribute to the understanding and design of care systems to serve people in treatment with OAT.

The 2016 *Surgeon General’s Report on Alcohol, Drugs and Health* [22] calls for an evidence-based approach to increase integration of substance use disorder treatment and general health care services, as have the Centers for Disease Control and Prevention [23, 24] and the Substance Abuse and Mental Health Services Administration [25]. The primary focus for integrated services nationally has been the addition of behavioral health into general health care services, in particular substance use screening and treatment in primary care settings. While this direction is critically important to expand both awareness of and access to substance use treatment and prevention services, our findings suggest that a “reverse integration” strategy, the incorporation of medical services into substance use treatment programs, offers another useful approach to integrated care for a patient population with historically low levels of engagement with preventive and routine health care [1, 4, 20].

Other examples of reverse integration models include screening as well as treatment services. Integration of HIV testing within substance use treatment programs, including methadone programs, has been shown to be feasible, acceptable to patients, and effective [26–28]. Participants attending community-based drug treatment programs were significantly more likely to receive their HIV results if testing was conducted on-site compared with a referral for off-site testing (*p* < .001, aRR = 4.52, 97.5% confidence interval = 3.57, 5.72) [28]. In a pilot study of HIV-infected PWID attending a syringe access program and not engaged in drug or HIV treatment at baseline (n = 13), on-site HIV treatment resulted in 85 and 54% of participants achieving viral suppression at 6 and 12 months, respectively [29]. Sylla et al. [30] proposed a model for integrated substance use, tuberculosis and HIV services that included screening and testing for each condition, co-location of services, provision of effective substance use treatment, enhanced monitoring for adverse events and cross-training of generalists and specialists in the target conditions in order to address the disparities in health care access and clinical outcomes for PWID. Smith-Rohrberg et al. [31] demonstrated that improved HIV virologic success (HIV viral load ≤ 400 copies/ml or a decrease from baseline viral load ≥ 1.0 log_10_ copies/ml) among PWID who received DAART at a mobile community health van providing syringe access services was associated with higher use of on-site medical and case management services compared with lower use of on-site services (89 vs. 64%, OR = 4.4, *p* = .03 for medical and 79 vs. 50%, OR = 4.0, *p* = .06 for case management services). They proposed that the proximity of services as well as strong interpersonal relationships between patients and staff may have contributed to successful treatment outcomes. Umbricht-Schneiter et al. [32] found that patients attending a methadone treatment program presenting with one of four key acute or chronic medical conditions (hypertension, purified protein derivative conversion, asymptomatic HIV infection and sexually transmitted infections) were more likely to receive medical care if treatment was onsite compared with referral for treatment (92 vs. 32%, *p* < .001).

In this study, we also found that a significantly higher proportion of HIV-infected women (96%) were virologically suppressed compared to men (71%), but that there was no sex difference in retention in HIV care. Historically, women have been less engaged in HIV care than men, which has been attributed to prioritization of family responsibilities, stigma, intimate partner violence, mental health and substance use disorders, and poverty [33]. However, 2011 United States data for all persons living with HIV from the National HIV Surveillance System and the Medical Monitoring Project showed no significant sex differences in viral suppression (32% for women, 29% for men) [7]. At OTOP, HIV-infected women demonstrated a significantly higher rate of treatment success (viral suppression) than men. Further research with OAT patients into the relationships of sex and housing stability and abstinence from alcohol and illicit substances, both associated with HIV treatment success [19, 34], may provide insight into the significantly higher viral suppression among women.

Our data also showed decreased viral suppression among OTOP patients <45 years old. Younger age is a known risk factor for poor engagement in care and worse treatment outcomes [7, 35]. In the SMILE collaborative, just 7% of HIV-infected youth between 12 and 24 years old obtained viral suppression [35]. Our data confirmed decreased viral suppression for our patients <45 years old. Young adults face particular challenges with engagement in care and medication adherence due to factors that include their stage of psychosocial and cognitive development, distrust of medical institutions and risk behavior [36]. At OTOP, our young adult patients are impacted by severe substance use disorders, social instability including homelessness, trauma and violence, and a lack of support during key developmental milestones. Our finding of worse treatment outcomes for our younger patients, though a small group, highlights the need to design integrated services that support engagement, adherence and ultimately viral suppression for this at-risk group.

The overall high levels of retention in care and viral suppression among OTOP patients living with HIV should also be viewed within the larger context of the wide availability of HIV primary care services across the city of San Francisco. Healthy San Francisco, a program of the San Francisco Department of Public Health and its community partners was started in 2007 to address the health care needs of uninsured residents [37, 38] and provides San Franciscans access to comprehensive preventive and primary care services, regardless of income and legal status. Health care access was further expanded in 2012 with Medicaid (MediCal) expansion and Covered California, the state’s health insurance marketplace. Policies supporting expanded access to care have facilitated linkage to HIV treatment for new patients entering OTOP who are not engaged in HIV care. OTOP is also the recipient of Ryan White Care Act funding that supports our efforts to improve engagement in care for our HIV-infected patients. However challenges to linkage and engagement in care remain, including patients with out-of-county MediCal, a history of distrust of medical systems, the stigmatization of substance use disorders, and recent federal threats to Medicaid expansion. Additional analyses that examine OTOP’s linkage data and explore the engagement status for HIV-infected patients at other methadone treatment programs in San Francisco would further our understanding of and guide interventions for these challenges.

Finally, HIV prevalence among OTOP patients (11%) may be higher than expected when compared with city and national prevalence among PWID alone (San Francisco 12%, USA 11%). The fact that OTOP enrolls persons with opioid use disorder who do not inject drugs in addition to people who do, as well as the anticipated HIV prevention impact of our city’s longstanding commitment to syringe service programs and a policy of substance use treatment on demand (factors associated with decreased risk for HIV transmission), suggests we might find a lower prevalence among OTOP patients. Possible explanations for OTOP’s HIV prevalence could be a high level of sexual risk behavior among our patient population or a greater tendency for people living with HIV and opioid use disorder to enter methadone treatment programs compared with their HIV uninfected counterparts. Further research is needed to explore these hypotheses.

This report has a number of limitations. Conducted as part of a quality improvement project, this descriptive study relied on retrospective chart reviews as data sources. Not only is our study design unable to establish causal relationships, but also the analysis is constrained by the types of variables available in the medical record. In addition, our analysis was limited by the group size of OTOP’s HIV-infected population in the study year. A challenge with a sample size of n = 68 is that fairly large differences need to be observed in order to reach statistical significance, which may be much larger than what we might think of as a clinically significant difference. This is evident in the differences we found in our analyses. An analysis with larger numbers of patients could be conducted by a consortium of methadone treatment programs offering integrated models of care to further examine our findings. Despite these limitations, our analysis provides valuable information about engagement in care for HIV-infected patients with opioid use disorder and a foundation from which to build individualized, targeted interventions.

## Conclusions

Retention in HIV care and viral suppression are high in this urban, publicly-funded, non-profit methadone clinic with integrated HIV primary care services. In addition to finding that female and older patients had significantly higher rates of viral suppression, this research supports the benefit of integrating HIV primary care and support services with methadone treatment services for this at-risk population.

## Abbreviations

EMR: electronic medical record
HIV: human immunodeficiency virus
OST: opioid substitution therapy
OTOP: Opiate Treatment Outpatient Program
SPSS: statistical package for the social sciences
VS: viral suppression
